# Supplementary material for: Association between oral microbiome and seven types of cancers in East Asian population: a two-sample Mendelian randomization analysis
Source: Front Mol Biosci. 2023 Nov 21;10:1327893. doi: 10.3389/fmolb.2023.1327893 (PMC10702768; doi:10.3389/fmolb.2023.1327893)
Supplement: Supplementary file 1 [file DataSheet1.ZIP › Supplementary Materials/MR plots of saliva/saliva═╝/Gastric cancer/pheno.917_to_gastric cancer_forest.pdf]

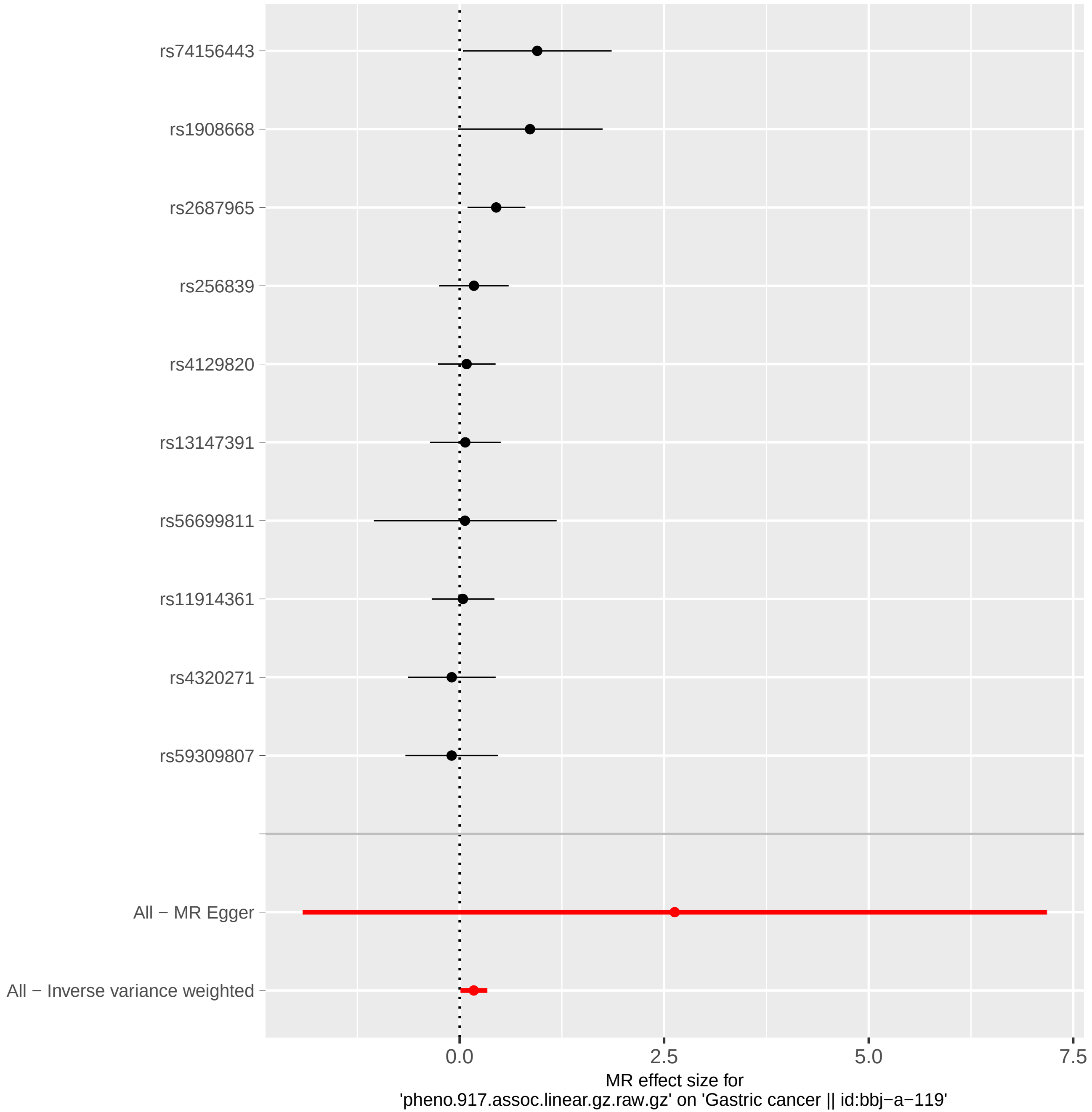
rs74156443
rs1908668
rs2687965
rs256839
rs4129820
rs13147391
rs56699811
rs11914361
rs4320271
rs59309807
All − MR Egger
All − Inverse variance weighted
0.0
2.5
5.0
7.5
MR effect size for
'pheno.917.assoc.linear.gz.raw.gz' on 'Gastric cancer || id:bbj−a−119'
